# Detection of breakage and impurity ratios for raw sugarcane based on estimation model and MDSC-DeepLabv3+

**DOI:** 10.3389/fpls.2023.1283230

**Published:** 2023-11-08

**Authors:** Xin Li, Zhigang Zhang, Shengping Lv, Tairan Liang, Jianmin Zou, Taotao Ning, Chunyu Jiang

**Affiliations:** College of Engineering, South China Agricultural University, Guangzhou, China

**Keywords:** raw sugarcane, breakage ratio, impurity ratio, estimation model, MDSC-DeepLabv3+

## Abstract

Broken cane and impurities such as top, leaf in harvested raw sugarcane significantly influence the yield of the sugar manufacturing process. It is crucial to determine the breakage and impurity ratios for assessing the quality and price of raw sugarcane in sugar refineries. However, the traditional manual sampling approach for detecting breakage and impurity ratios suffers from subjectivity, low efficiency, and result discrepancies. To address this problem, a novel approach combining an estimation model and semantic segmentation method for breakage and impurity ratios detection was developed. A machine vision-based image acquisition platform was designed, and custom image and mass datasets of cane, broken cane, top, and leaf were created. For cane, broken cane, top, and leaf, normal fitting of mean surface densities based on pixel information and measured mass was conducted. An estimation model for the mass of each class and the breakage and impurity ratios was established using the mean surface density and pixels. Furthermore, the MDSC-DeepLabv3+ model was developed to accurately and efficiently segment pixels of the four classes of objects. This model integrates improved MobileNetv2, atrous spatial pyramid pooling with deepwise separable convolution and strip pooling module, and coordinate attention mechanism to achieve high segmentation accuracy, deployability, and efficiency simultaneously. Experimental results based on the custom image and mass datasets showed that the estimation model achieved high accuracy for breakage and impurity ratios between estimated and measured value with *R^2^* values of 0.976 and 0.968, respectively. MDSC-DeepLabv3+ outperformed the compared models with mPA and mIoU of 97.55% and 94.84%, respectively. Compared to the baseline DeepLabv3+, MDSC-DeepLabv3+ demonstrated significant improvements in mPA and mIoU and reduced Params, FLOPs, and inference time, making it suitable for deployment on edge devices and real-time inference. The average relative errors of breakage and impurity ratios between estimated and measured values were 11.3% and 6.5%, respectively. Overall, this novel approach enables high-precision, efficient, and intelligent detection of breakage and impurity ratios for raw sugarcane.

## 1 Introduction

Sugarcane is an important raw material for the sugar industry worldwide. In China, sugarcane-based sugar production reached 4.6 million tons in 2022, which is 4.3 times that of beet sugar (National Development and Reform Commission, 2023). In recent years, the use of machine-harvested sugarcane has been steadily increasing, with plans to reach 30% of total sugarcane harvest in China by 2025 (Chinese government website, 2018). Machine harvesting significantly improves efficiency and reduces labor intensity; however, it also leads to higher ratios of broken cane and impurities such as top, leaf, which can negatively impact the yield of the sugar manufacturing process. As a result, the breakage and impurity ratios are crucial indicators for assessing the quality and pricing of raw sugarcane in practice, and determining these two ratios is indispensable for sugar refineries. Unfortunately, the commonly used manual sampling approach for detecting breakage and impurity ratios brings several issues, including strong subjectivity, low efficiency, and significant result discrepancies.

To address the aforementioned problem, an estimation model was established, and machine vision technology was employed to provide a more objective, efficient, accurate, and intelligent approach for quantifying the cane, broken cane, and impurities, as well as the ratios of breakage and impurity. This enables seamless integration with the sugarcane harvesting and sugar processing stages. Both cane and broken cane can be used as raw materials, but broken cane is considered in mass deduction by sugar refineries because it results in the loss of sugar content and impacts the quality of the final sugar product. The sugarcane top, leaf, root, sand, gravel, and soil and so forth are collectively referred to as impurities (Guedes and Pereira, 2018). Adjusting the height between the harvester’s cutting device and the ridge surface will reduce the introduction of sand, gravel, and soil during sugarcane harvesting. Furthermore, when the mechanical harvester operates smoothly and adheres to specifications, it noticeably decreases the levels of mud, stone, and cane root (Xie et al., 2018). Mechanical removal methods, such as vibration, can often be used to screen out the sand, gravel, and soil (Martins and Ruiz, 2020). However, the top, leaf and cane root are unavoidable impurities as they are naturally part of each sugarcane stem (de Mello et al., 2022). Regarding cane root, object detection can be utilized to count its quantities. Combining this with the average weight of the cane root helps predict the mass of root impurity after excluding sand, gravel and soil. Based on the quality detection practice of sugar refineries, the four categories of cane, broken cane, top, and leaf are selected as the detection objects in this study.

Estimation models and machine vision technology have been widely used for the detection and monitoring of impurities in grain crops such as rice, wheat, and corn. For example, Chen et al. (2020) used morphological features and a decision tree for the classification of rice grains and impurities with 76% accuracy to optimize combine harvester parameters. Liu et al. (2023) proposed a NAM-EfficientNetv2 lightweight segmentation approach for rapid online detection of rice seed and impurities in harvesters, achieving high evaluation index F1 scores of 95.26% and 93.27% for rice grain and impurities, respectively. To improve accuracy in wheat and impurity recognition, Shen et al. (2019) constructed a dataset and trained a recognition model called WheNet based on Inception_v3, achieving a recall rate of 98% and an efficiency of 100ms per image. Chen et al. (2022) designed a vision system based on DeepLabv3+ to identify seeds and impurities in wheat, obtaining mean pixel accuracy (mPA) values of 86.86% and 89.91% for grains and impurities, and mean intersection over union (mIoU) scores of 0.7186 and 0.7457, respectively. For the detection of impurities in the corn deep-bed drying process, Li et al. (2022) employed a multi-scale color recovery algorithm to enhance images and eliminate noise. They used HSV color space parameter thresholds and morphological operations for segmentation and achieved F1 scores of 83.05%, 83.87%, and 87.43% for identifying broken corncob, broken bract, and crushed stone, respectively. Liu et al. (2022) developed a CPU-Net semantic segmentation model based on U-Net, incorporating the convolutional block attention module (CBAM) and pyramid pooling modules to improve segmentation accuracy for monitoring corn kernels and their impurities. They established a mass-pixel linear regression model to calculate the kernel impurity rate and experimental results demonstrated that CPU-Net outperforms other comparative approaches with average mIoU, mPA, and inference time scores of 97.31%, 98.71%, and 158.4ms per image, respectively. The average relative error between the impurity rate obtained by the model and manual statistics was 4.64%.

Detection of impurities in cash crops such as soybean, cotton, and walnut during harvesting or processing has also been extensively studied in recent years. Momin et al. (2017) used HSI to segment the image background of soybean with three categories of impurities. They employed various image processing techniques, such as median blur, morphological operations, watershed transformation, projection area-based analysis, and circle detection, for feature recognition of soybean and impurities. The experimental results showed pixel accuracy of 96%, 75%, and 98% for split bean, contaminated bean, and defective bean, and stem/pod, respectively. Jin et al. (2022) developed an improved UNet segmentation model to address issues of soybean sticking, stacking, and complex semantics in images. The experimental results demonstrated comprehensive evaluation index values of 95.50%, 91.88%, and 94.34% for complete grain, broken grain, and impurity segmentation, respectively, with a mIoU of 86.83%. The field experiment indicated mean absolute errors of 0.18 and 0.10 percentage points for fragmentation and impurity rate between the model-based value and the measured value, respectively. For real-time detection of impurity ratio in cotton processing, Zhang et al. (2022) utilized the enhanced Canny algorithm to segment cotton and its impurities. They employed YOLOv5 to identify the segmented objects and determine their respective categories. They also developed an estimation model for the impurity ratio based on segmented volume and estimated mass and utilized a multithread technique to shorten the processing time, achieving a 43.65% reduction compared to that of a single thread. To improve the recognition accuracy of white and near-cotton-colored impurities in raw cotton, Xu et al. (2023) proposed a weighted feature fusion module and a decoupled detection strategy to enhance the detection head of YOLOv4-tiny. The proposed method decreased computation during the inference process, boosted the speed of inference, and enhanced the accuracy of cotton impurity localization. Experimental results showed a respective increase of 10.35% and 6.9% in mAP and frames per second (FPS) compared to the baseline YOLOv4-tiny. The detection accuracy of white and near cotton-colored impurities in raw cotton reached 98.78% and 98%, respectively. To achieve real-time segmentation of juglans impurity, Rong et al. (2020) proposed a hybrid approach by combining a segmentation model based on a multi-scale residual full convolutional network and a classification method based on a convolutional network. The proposed method accurately segmented 99.4% and 96.5% of the object regions in the test and validation images, respectively, with a segmentation time of within 60ms for each image. Yu L. et al. (2023) presented an improved YOLOv5 with lower parameters and quicker speed for walnut kernel impurity detection by incorporating target detection layers, CBAM, transformer-encoder, and GhostNet. The results indicated a mAP of 88.9%, which outperformed the baseline YOLOv5 by 6.7%.

In recent years, researchers have also achieved notable progress in the field of impurity detection in sugarcane. Guedes and Pereira (2019) constructed an image dataset comprising 122 different combinations of sugarcane stalk, vegetal plant part, and soil to evaluate the impurity amount. They converted color samples into color histograms with ten color scales and employed three classifiers, namely soft independent modeling of class analogy, partial least squares discriminant analysis (PLS-DA), and *k* nearest neighbors (KNN), to classify cane and its impurities. Guedes et al. (2020) further proposed an analytical method using artificial neural networks (ANNs) combined with the ten color histograms to predict the content of sugarcane in the presence of impurities. The experimental results demonstrated correlation coefficients of 0.98, 0.93, and 0.91 for the training, validation, and test sets, respectively. Aparatana et al. (2020) employed principal component analysis (PCA), PLS-DA, and support vector machine (SVM) to classify and differentiate sugarcane and impurities, including green leaf, dry leaf, stone, and soil, based on their spectral information. The research findings indicated that PCA, PLS-DA, and SVM achieved classification rates of 90%, 92.9%, and 98.2%, respectively. Dos Santos et al. (2021) used a similar mechanism by combining ten color histograms and ANNs to classify raw sugarcane. They achieved 100% accurate classification for two ranges of raw sugarcane in the samples, from 90 to 100 wt% and from 41 to 87 wt%. However, these studies mentioned above recognize raw sugarcane and impurities based on their color features, making it difficult to differentiate objects with inter-class similarity, such as sugarcane top and leaf, which have similar color features at the pixel level. Additionally, these methods may not be suitable for practical situation with multiple combinations of impurities in arbitrary proportions, which present significant challenges in building samples with a vast combination of weight percentages of impurities.

From the perspective of recognition tasks, the aforementioned studies can be categorized into three types: image classification, object detection, and semantic segmentation. Image classification-based approaches (Momin et al., 2017; Guedes and Pereira, 2019; Shen et al., 2019; Aparatana et al., 2020; Chen et al., 2020; Guedes et al., 2020; Dos Santos et al., 2021; Li et al., 2022) cannot capture pixel-level information for subsequent construction of a mass-pixel fitting model. Object detection can be utilized for real-time classification and localization of crops and impurities (Zhang et al., 2022; Xu et al., 2023; Yu J. et al., 2023), but they still cannot support subsequent mass estimation based on pixels of detected objects. Semantic segmentation, on the other hand, enables pixel-wise classification of an image and facilitates the precise determination of the number of pixels and their respective categories in a specific region. Mass-pixel fitting models can be established by combining the number of pixels and the actual mass of each category of object (Rong et al., 2020; Chen et al., 2022; Jin et al., 2022; Liu et al., 2022; Liu et al., 2023), thus supporting the quantitative analysis of the quality of the detected objects. In order to quantify the ratio of breakage and impurity in raw sugarcane, semantic segmentation technology was utilized to abstract the of raw sugarcane and impurities in this study. However, the aforementioned approaches and findings are difficult to be directly applied to the detection of sugarcane and impurities in this study. Firstly, there is currently a lack of image databases that include raw sugarcane and impurities. Secondly, the estimation models developed in the above studies are only suitable for relatively stable scenarios of surface density (mass/pixel) for each detection category. However, the surface density of broken cane varies significantly due to different degrees of breakage, and the residual leaf at the top of the cane is scattered, resulting in a more varied surface density. Therefore, it is necessary to establish a corresponding image dataset and segmentation model for the detection of raw sugarcane and impurities and build new estimation model for quality evaluation based on segmented pixels.

Popular and widely applied deep learning (DL)-based semantic segmentation approaches have achieved excellent results in image processing in agriculture (Luo et al., 2023). Among these approaches, end-to-end semantic segmentation models like FCN, UNet, PSPNet, and DeepLabv3+ have demonstrated good performance with simple structures. DeepLabv3+ in particular has gained significant popularity and has been extensively enhanced due to its exceptional segmentation accuracy, making it a widely practiced and verified model in agricultural applications. For instance, Wu et al. (2021) developed an enhanced version of DeepLabv3+ to segment abnormal leaves in hydroponic lettuce. Peng et al. (2023) constructed an RDF-DeepLabv3+ for segmenting lychee *stem*. Zhu et al. (2023) proposed a two-stage DeepLabv3+ with adaptive loss for the segmentation of apple leaf disease images in complex scenes. Wu et al. (2023) utilized Deeplabv3+ and post-processing image analysis techniques for precise segmentation and counting of banana bunches. Their findings indicated that DeepLabv3+-based segmentation models can effectively perform pixel-level segmentation of crop objects, and the segmentation effects were superior to those of compared approaches. In this study, DeepLabv3+ was adopted for the semantic segmentation of raw sugarcane and impurities, and efforts were made to further improve its segmentation accuracy, reduce parameters, and optimize inference time.

This study aims to address the detection of breakage and impurity ratios in raw sugarcane. The specific research content of this study includes: (1) Designing a machine vision-based acquisition platform for online image collection of raw sugarcane (cane, broken cane) and impurities (top, leaf). Custom datasets of masses and corresponding images were constructed. (2) Establishing a normal fitting model to determine the mean surface density of each class based on measured masses and extracted pixels. Additionally, an estimation model was developed to assess the ratios of breakage and impurity using the estimated mass of each class, along with their pixels and fitted mean surface density. (3) Developing a MDSC-DeepLabv3+ model for accurate segmentation of raw sugarcane and impurity pixels based on DeepLabv3+. The model was further improved by incorporating improved MobileNetv2, atrous spatial pyramid pooling (ASPP) with deepwise separable convolution (DSC) and strip pooling (SP) named ASPP_DS, and coordinate attention (CA) mechanism to enhance segmentation accuracy, reduce parameters, and optimize inference time. (4) Conducting experiments to verify the accuracy of the proposed estimation model in assessing breakage and impurity ratios, and evaluate the capability of MDSC-DeepLabv3+ in rapidly and accurately identifying the pixels of cane, broken cane, top, and leaf. Comprehensive experimental results show that the average relative errors of breakage and impurity ratio between predicted values and measured values are low. These findings have significant implications for the development of intelligent detection and cleaning system for sugarcane impurity.

## 2 Materials and methods

### 2.1 Raw sugarcane and impurity dataset construction

#### 2.1.1 Detection device design

**Figure 1:**
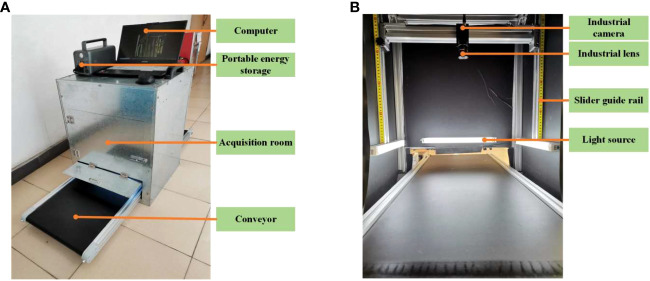
Machine vision acquisition platform. **(A)** Acquisition device structure. **(B)** Acquisition room.

In order to provide a stable environment and meet the continuous image acquisition requirements that align with the raw sugarcane convey process in the sugar refinery, a dedicated platform for image acquisition of raw sugarcane and impurities was designed, as shown in **Figure 1A**. The platform mainly consists of portable energy storage, an acquisition room, a light source, an image acquisition module, a computer, and a motion assistance module.

The portable energy storage is used to supply power to the platform, especially in situations where electricity supply is limited. The interior of the image acquisition room, as depicted in **Figure 1B**, is covered with black matte paper to create a diffused lighting environment. Additionally, four magnetic base LED light bars are strategically placed around the room to ensure consistent illumination for the image acquisition module. The image acquisition module comprises an industrial camera and an industrial lens. The computer is connected to the image acquisition module via a USB 3.0 interface, which facilitates image storage and processing. The motion assistance module is composed of a conveyor, a cross beam guide rail, and a pair of vertical slider guide rails with self-locking function. The conveyor simulates the transmission of raw sugarcane before entering the pressing workshop. The vertical slider guide rails, equipped with scale markings, support and allow for adjustment of the cross beam guide rail where the camera is mounted. This feature enables easy adjustment of the camera’s field of view and ensures the stability of the image acquisition module.

**Figure 2:**
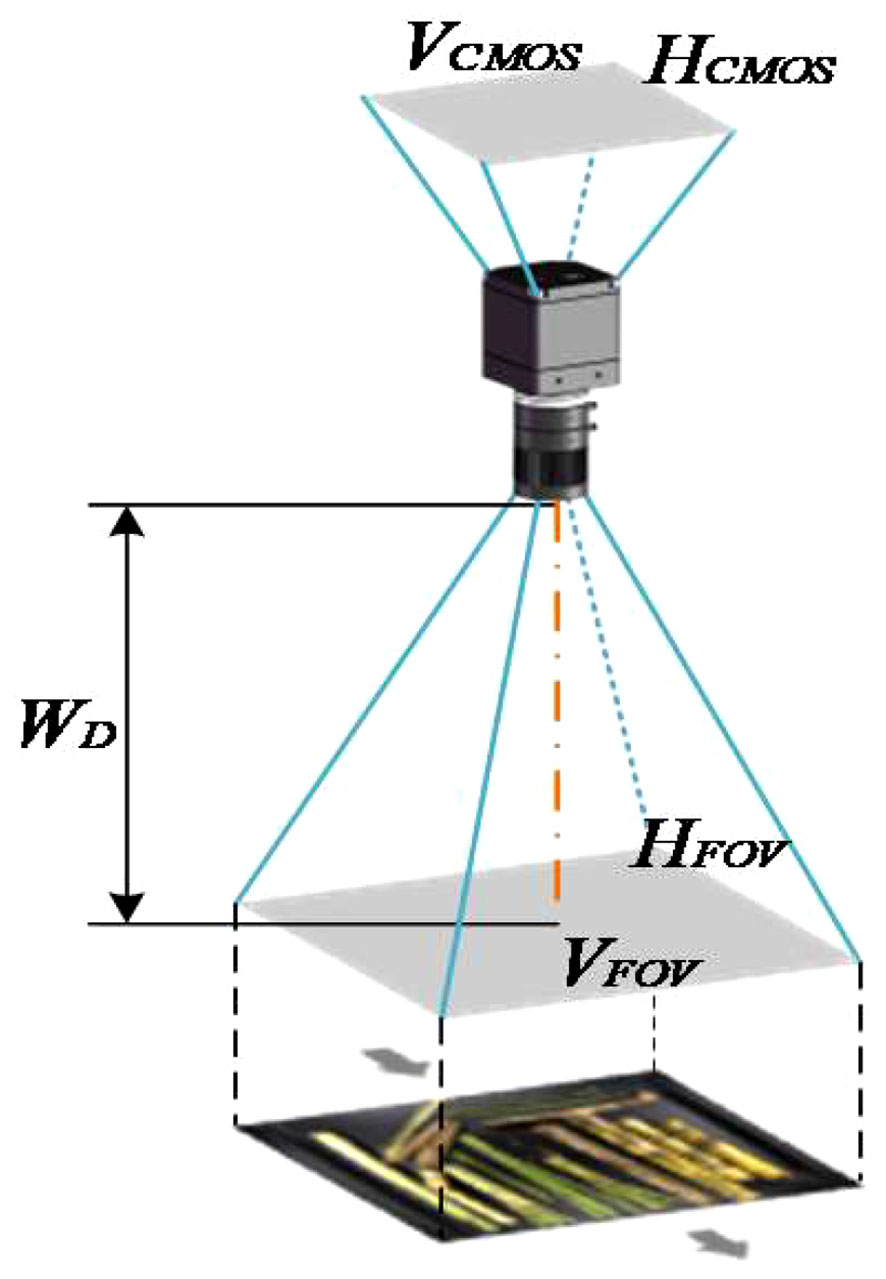
Imaging principle in this acquisition platform.


**Table 1** shows the model parameters of the main components of the acquisition platform. The conveyor belt speed is determined based on sugar refinery practice and is measured in meters per second (m/s). The dimensions of the indoor acquisition room are set according to the requirements, with horizontal (*H_FOV_*) and vertical (*V_FOV_*) dimensions are set to the belt width of 450mm and indoor length of 600mm, respectively. The selected industrial camera has a horizontal (*H_CMOS_*) and vertical (*V_CMOS_*) size of the image sensor as 7.6×5.7mm, and the working distance (*W_D_*) is set to 490mm considering the inner height of the acquisition room. The imaging principle of this acquisition platform is illustrated in **Figure 2**. Using the imaging principle and the dimensions of *H_CMOS_*, *V_CMOS_* and *W_D_*, the field of view can be determined using Eq.(1).

**Table 1:** Main components of the acquisition platform.

Components	Parameters	Components	Parameters
Acquisition room	Indoor space 600mm×500mm×700 mm	Slider guide rail	SGR15N-500mm×2
Industry camera	MV-CA020-10UC with 89.1fps@1624×1240, image sensor size 7.6×5.7mm	Computer	AMD Ryzen7 5800H GeForce GTX 1650
Industry lens	MVL-MF0828M-8MP	Portable energy storage	72000mAh/3.2V
Light source	3600Lux×4	Conveyor	2000mm×450mm×100mm,1.5m/s, ≤20kg

(1) $$f/W_{D}=V_{CMOS}/V_{FOV}=H_{CMOS}/H_{FOV}$$

As a result, the focal length is determined by *f* = *W_D_* × (*V_CMOS_*/*V_FOV_*) = 490 × (7.6/3450) = 8.27mm, and the MVL-MF0828M-8MP industry lens is selected.

#### 2.1.2 Image and mass data acquisition

**Figure 3:**
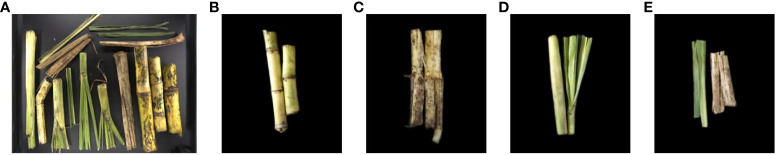
Acquisition materials and segmentation classes. **(A)** Original image, **(B)** Cane, **(C)** Broken cane, **(D)** Top, **(E)** Leaf.

The image and mass acquisition of raw sugarcane and impurities took place in the sugarcane unloading workshop of Junshi sugar refinery in Jijia Town, Leizhou City, Guangdong Province. The data collection period started from the middle of February to the end of the month in 2023, coinciding with the local sugarcane harvesting season. For this study, large-scale cultivated sugarcane variety “Yuetang 159” was selected. The raw sugarcane samples were randomly collected from different machine-harvested vehicles at various time intervals throughout the day using a loader. These samples were then manually placed on the conveyor belt of the acquisition platform for image collection. In total, 910 RGB 8-bit photos with jpg format and a resolution of 1624×1240 were captured. Each image contains four categories: cane, broken cane, top, and leaf, as shown in **Figure 3**. Following the image capturing process, 300 samples of raw sugarcane and impurities were randomly selected from the collected images. Each category of material in these samples was weighed using a calibrated electronic scale with a precision of 0.01g, and their masses were measured in grams (g).

#### 2.1.3 Image labeling and dataset augmentation

The original dataset consists of 910 images containing cane, broken cane, top, leaf, and the background. These images were manually labeled and colored using the image annotation tool Labelme. The labeled regions of the five classes of objects were used to evaluate the training loss of intersection over union (IoU) between predicted bounding boxes and ground truth. The RGB values for cane, broken cane, top, and leaf were set to [128,0,0], [0,0,128], [0,128,0], and [128,128,0], respectively, while the background was set to [0,0,0]. To ensure model performance validation and testing, the dataset was randomly divided into training (546 images), validation (182 images), and test sets (182 images) with a ratio of 6:2:2.

**Figure 4:**
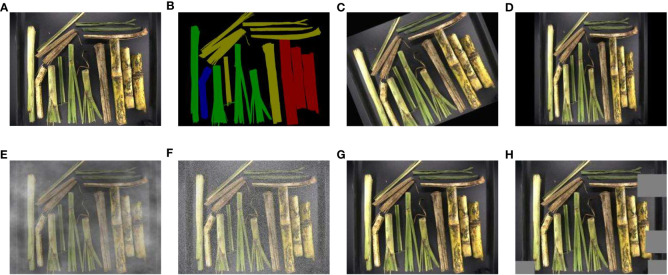
Augmented image samples and image label. **(A)** Original image, **(B)** Ground truth, **(C)** Random rotation, **(D)** Affine transformation, **(E)** Fogging, **(F)** Gaussian noise, **(G)** Median filtering, **(H)** Cutout.

In order to improve the generalization of the model, data augmentation techniques were applied to the training, validation, and test sets separately. Techniques such as random rotation, affine transformation, fogging, Gaussian noise, median filtering, and cutout were used to enhance the original images. After augmentation, the images were checked and corrected using Labelme to ensure accurate labeling of each class in every image. The annotated images were stored in the PASCAL VOC format and named Raw Sugarcane and Impurity (RSI). The label counting algorithm was used to calculate the number of labels in the RSI images, and the corresponding statistics are shown in **Table 2**. The dataset demonstrates a relatively balanced distribution of samples across each class. Examples of the original annotated images and augmented images can be observed in **Figure 4**.

**Table 2:** Statistic of Raw Sugarcane and Impurity (RSI) dataset.

Dataset	Training dataset	Validation dataset	Test dataset	Complete dataset
Images	5460	1820	1820	9100
Cane labels	16882	4151	3850	24883
Broken cane labels	13735	3310	3410	17045
Top labels	15903	4071	4390	24364
Leaf labels	17234	4015	3830	25079

### 2.2 Estimation model establishment

#### 2.2.1 Surface density distribution analysis

In general, previous estimation models that are based on image pixels for assessing the mass of crops (such as wheat, corn, and soybean) often assume that the surface density (mass/pixel) of each crop category remains stable across different images (Chen et al., 2022; Jin et al., 2022; Liu et al., 2022). However, when it comes to broken cane and impurities, their surface density can vary significantly in different images. Therefore, before building the estimation model, it is essential to analyze the surface density distributions of cane, broken cane, top, and leaf separately. This analysis will help to account for the variation in surface density and ensure more accurate estimation for breakage and impurity ratios in raw sugarcane.

**Figure 5:**
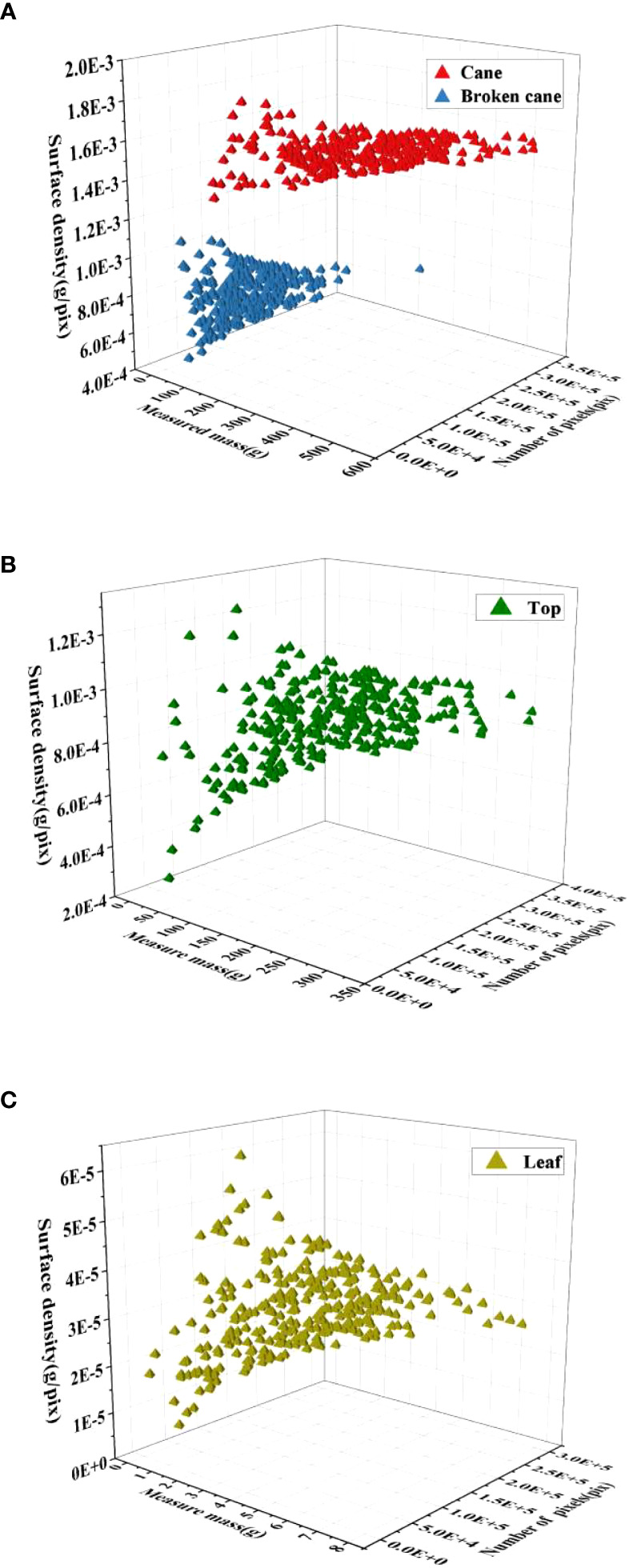
Spatial distribution of surface density for the 4 classes in RSI. **(A)** Raw sugarcane, **(B)** Top, **(C)** Leaf.

The analysis of surface density distribution was conducted using 300 samples of mass data and the corresponding images for each category. The OpenCV threshold function was utilized to count the number of pixels in each category. Let *P_C_*, *P_B_*, *P_T_* and*P_L_* represent the number of pixels of cane, broken cane, top, and leaf in each image sample, respectively, and their corresponding masses are denoted as *M_C_*, *M_B_*, *M_T_* and *M_L_*, respectively. The spatial distribution of the surface density for raw sugarcane, including cane and broken cane, as well as the top and leaf, is presented in **Figure 5**. Based on the surface density distribution of raw sugarcane in **Figure 5A**, it can be observed that the surface density of cane fluctuates less and is more concentrated. The surface density of broken cane is approximately half of that of cane, and the data is scattered. **Figures 5B, C** illustrate that the surface density distribution of top and leaf is more scattered compared to broken cane.

**Figure 6:**
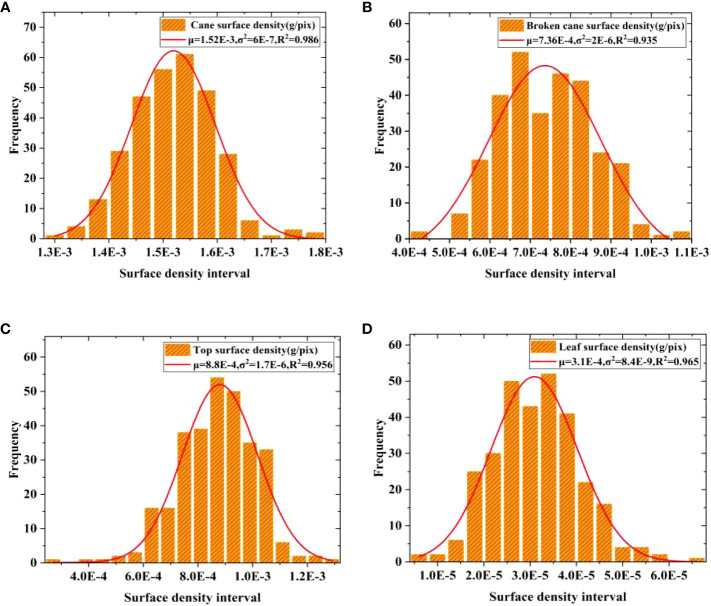
Gaussian distribution fitting of surface density. **(A)** Cane, **(B)** Broken cane, **(C)** Top, **(D)** Leaf.

To address the scattered surface density of broken cane, top, and leaf, a Gaussian distribution probability density function was used to fit the frequency histograms of surface density for each category. The mean surface density *μ* for each category was then obtained through the fitting process, and the results are demonstrated in **Figure 6**. It can be observed that all fitting coefficients *R* ^2^ are greater than 0.95, indicating high fitting accuracy.

The fitting results showed that the mean surface density of cane, broken cane, top, and leaf are *μ_c_* = 1.52E-3, *μ_B_* 7.4E-4, *μ_T_* = 8.8E-4 and *μ_L_* = 3E-5 with unit g/pix, respectively. Moreover, it is evident that the mean value of cane *μ_c_* is approximately twice the mean value of broken cane *μ_B_* and top surface density *μ_T_*, and *μ_c_* is more than fifty times *of μ_L_*. The mass error of leaf has little effect on the overall mass error. Therefore, when establishing the estimation model, the accuracy of the estimated mass of cane should be ensured first, followed by broken cane, top, and finally leaf. This approach is consistent with the low deduction percentage setting (as low as 0.2%) employed by sugar refineries for leaf impurities.

#### 2.2.2 Fitting and estimation model establishment

On the basis of the mean values of surface density given in **Figure 6**, the estimated mass of cane *M’_C_*, broken cane *M’_B_*, top *M’_T_*, and leaf *M’_L_* based on their pixels can be expressed as follows:


(1) $${M^{\prime }}_{C}=\mu_{C}\times P_{C}=1.52E-3P_{C}$$

(2) $${M^{\prime }}_{B}=\mu_{B}\times P_{B}=7.4E-4P_{B}$$

(3) $${M^{\prime }}_{T}=\mu_{T}\times P_{T}=8.8E-4P_{T}$$

(4) $${M^{\prime }}_{L}=\mu_{L}\times P_{L}=3E-5P_{L}$$

**Figure 7:**
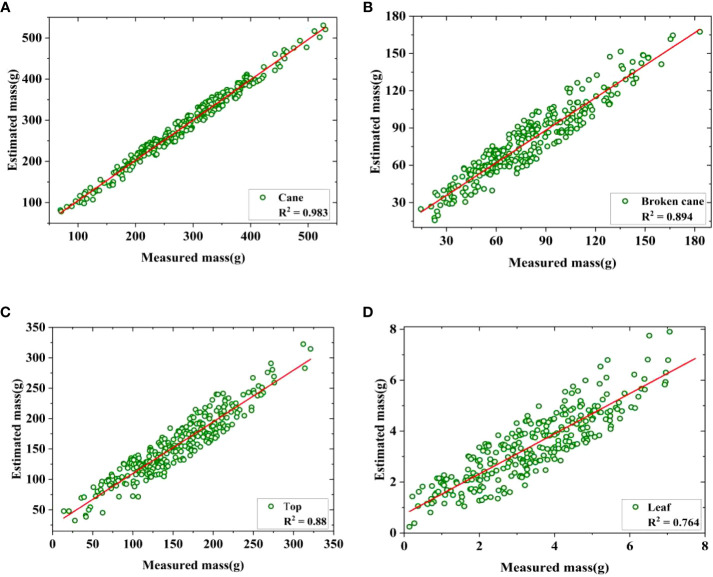
Regression of estimated and measured mass. **(A)** Cane, **(B)** Broken cane, **(C)** Top, **(D)** Leaf.

Furthermore, a linear regression of the estimated and measured mass was conducted to validate the accuracy of the mass estimation model defined by Eq.(1)-(4). Based on the distribution characteristics shown in **Figure 6**, a total of 285 mass data of cane, broken cane, top, and leaf within a 95% confidence interval were selected for fitting, and the fitting results were presented in **Figure 7** and **Table 3**. It can be seen that the measured mass of the cane is highly correlated with the estimated mass with an *R^2^* value of 0.983. This indicates that the linear regression model is capable of explaining the numerical relationship between the measured mass and the estimated mass of the cane. The *R^2^* value for broken cane and top are 0.894 and 0.88, respectively, demonstrating the regression model’s good fitting capability. The *R^2^* value for the leaf is 0.764 suggesting that the model can still adequately fit the relationship between the measured mass and the estimated mass. In addition, the results of ANOVA in **Table 3** indicate that the significance *F*<0.01 between estimated cane, broken cane, top, and leaf and their measured values proves a high correlation.

**Table 3:** Analysis of Variance (ANOVA) of estimated and measured mass. .

Category		DF	Square sums	Mean square	F	Significance F
Cane	Regression analysis	1	2340192.15697	2340192.15697	16820.25846	4.23041E-254
	Residual	283	39373.61497	139.12938		
	Total	284	2379565.77194			
Broken cane	Regression analysis	1	225202.9665	225202.9665	2390.43448	4.97988E-140
	Residual	283	26661.44583	94.21006		
	Total	284	251864.41233			
Top	Regression analysis	1	656015.70993	656015.70993	2055.21929	8.58987E-132
	Residual	283	90332.18347	319.19499		
	Total	284	746347.8934			
Leaf	Regression analysis	1	431.20971	431.20971	915.53104	1.07792E-90
	Residual	283	133.29133	0.47099		
	Total	284	564.50104			

Based on the mass of each category, the ratios of breakage (*R_B_*) and impurity (*R_I_*) *is* defined as:


(5) $$\begin{array}{l} R_{B}=\frac{M_{B}}{M_{C}+M_{B}}\times 100\%\\ =\frac{7.4E-4\;\times \;P_{E}}{1.52E-3\times P_{c}+7.4E-4\times P_{E}}\times 100\% \end{array}$$

(6) $$\begin{array}{l} R_{I}=\frac{M_{T}\;+\;M_{L}}{M_{C}+M_{B}+M_{T}+M_{L}}\times 100\%\\ =\frac{8.8E-4\times P_{T}\;+\;3E-5\times P_{L}}{1.52E-3\times P_{c}+7.4E-4\;\times \;P_{E}+8.8E-4\times P_{T}\;+\;3E-5\times P_{L}}\times 100\% \end{array}$$

Where *M_C_*, *M_B_*, *M_T_* and *M_L_* is the mass of cane, broken cane, top and leaf in an image sample. The estimated breakage and impurity ratios R'_B_ and R'_I_ can also be determined by replacing M_C_, M_B_, M_T_ and M_L_ in Eq.(5)-(6) with estimated mass M'_C_, M'_B_, M'_T_ and M'_L_. Thereby Eq.(5)-(6) can be taken as the estimation model for breakage and impurity ratios.

### 2.3 Raw sugarcane and impurity segmentation model development

#### 2.3.1 MDSC-DeepLabv3+ framework

In order to facilitate the *M'_C_, M'_B_, M'_T_*, *M'_L_*, *R'_B_* and *R'_I_* calculation, a segmentation model, MDSC-DeepLabv3+, was developed for the intelligent extraction of pixels of cane *P_C_*, broken cane *P_B_*, top *P_T_*, and leaf *P_L_* in each image sample. MDSC-DeepLabv3+ is an improvement upon the DeepLabv3+. The DeepLabv3+ comprises two modules: an encoder and a decoder (Chen et al., 2018). In the encoder, the Xception backbone is used to extract input image features, resulting in two effective feature maps. One of the feature map undergoes processing through atrous spatial pyramid pooling named ASPP, and is then using a 1×1 standardization convolution for the fused features from ASPP. This produces high-level features that are subsequently fed into the decoder. The other feature map directly outputs to the decoder. The ASPP is composed of a 1×1 standardization convolution, three 3×3 depthwise separable convolutions named DSC with varying dilation rates (6, 12, and 18), and an average pooling layer. These convolutions generate feature maps at four different scales, which are stacked along the channel dimension.

In the decoder, the low-level features obtained from the Xception backbone first undergo 1×1 convolution to reduce the number of channels. Meanwhile, the high-level features from the encoder are bilinearly upsampled by a factor 4 to improve the image resolution. Afterwards, the 1×1 convoluted low-level features are fused with the upsampled high-level features, and a 3×3 DSC is utilized to extract information from the fused features, followed by another bilinear upsampling by a factor 4. Previous studies have demonstrated the effective use of DeepLabv3+ in agricultural fields, such as fruit picking, crop disease and pest, and field road scenes (Wu et al., 2021; Peng et al., 2023; Yu J. et al., 2023).

**Figure 8:**
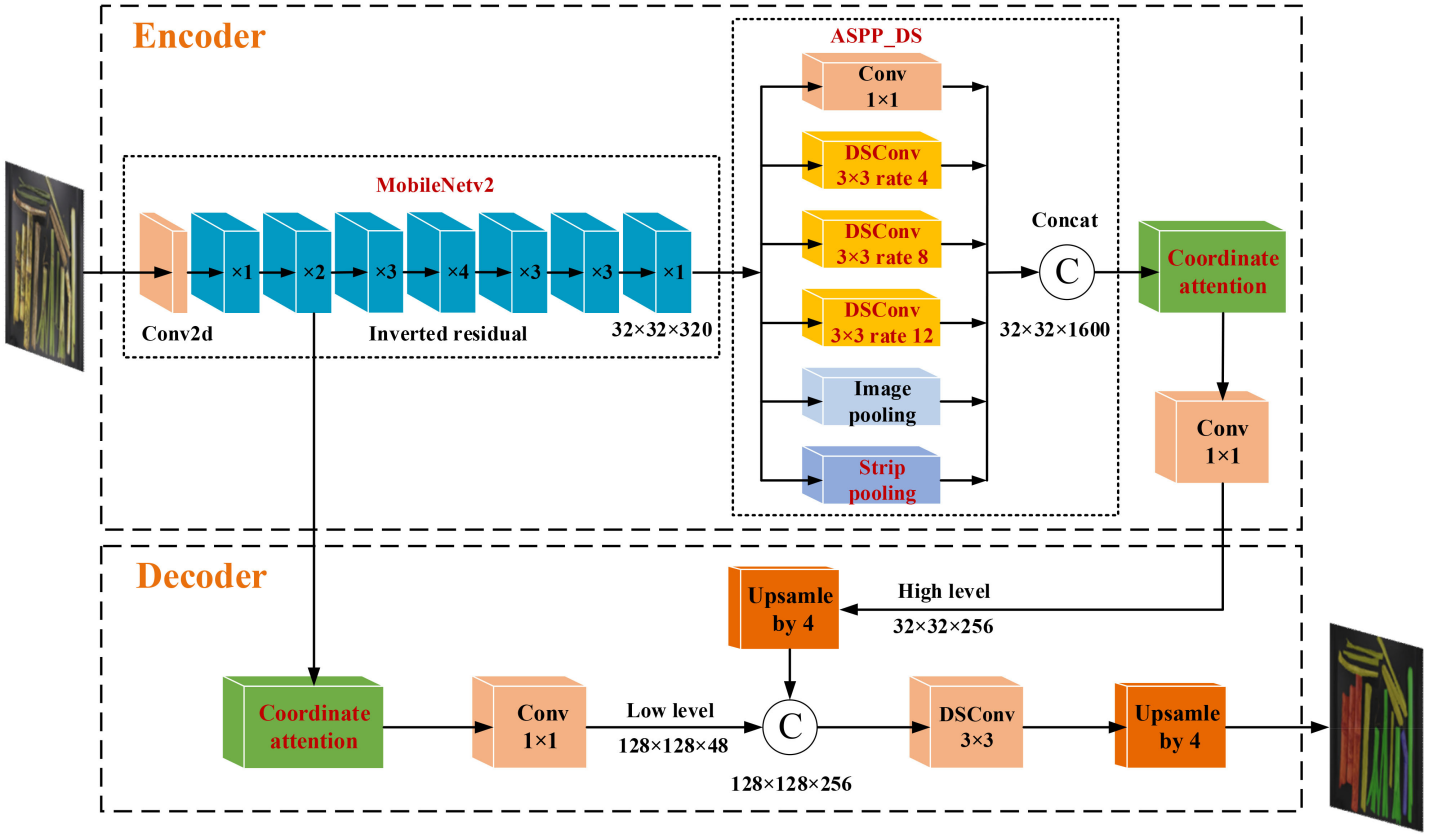
Framework of MDSC-DeepLabv3+.

To enhance both the accuracy and deployability of the model, as well as reduce inference time, various improvements including improved MobileNetv2, ASPP_DS module and CA mechanism were introduced in this study. First, the atrous convolution was employed to optimize the MobileNetv2, and Xception was replaced by the improved MobileNetv2 in DeepLabv3+. In the MobileNetv2, dilated convolution was incorporated into the last two layers by increasing the kernel size, thus expanding the receptive field. This enhancement allows the network to better perceive surrounding information without significantly increasing computational complexity or compromising the resolution of the feature maps. Then, the dilation rates in the ASPP module were adjusted as 4, 8, and 12, and a strip pooling layer was added parallel to DSC to build a module named ASPP_DS. Module ASPP_DS can reduce the model parameters and establish long-range dependencies between regions distributed discretely, and focus on capturing local details. ASPP employs diverse padding and compact dilation strategies to extract receptive fields at various scales, effectively capturing information from both multi-scale contexts and small objects. Additionally, ASPP integrates a parallel strip pooling layer with elongated and narrow pooling kernels to grasp local contextual details in both horizontal and vertical spatial dimensions. This approach helps in reducing interference from unrelated regions in label prediction results. Finally, CA was appended to the output of MobileNetv2 and ASPP_DS separately, that allows the model to acquire weight information from the dimensions of feature channels and effectively leverage positional data. This incorporation enables the accurate capture of spatial relationships and contextual information of the target, thereby enhancing training efficiency. The enhanced version of DeepLabv3+ is denoted as MDSC-DeepLabv3+. The overall framework of MDSC-DeepLabv3+ is depicted in **Figure 8**.

#### 2.3.2 Improved MobileNetv2

**Figure 9:**
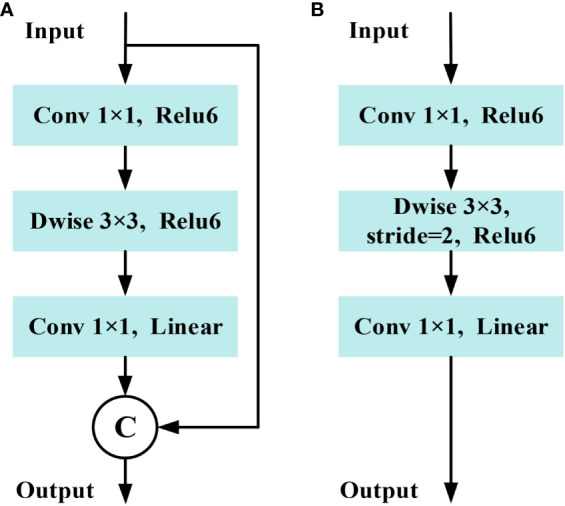
Structure of inverted residual block in MobileNetv2. **(A)** Stride=1 block. **(B)** Stride=2 block.

The basic structure unit of MobileNetv2 is the inverted residual block (IRB), which mainly consists of dimensionality expansion, feature extraction and dimensionality compress three main steps. The MobileNetv2 employs 3×3 depthwise convolution (Dwise) and 1×1 convolution to construct two IRBs with s= 1, s=2 (Sandler et al., 2018). In cases where the stride is equal to 1 and the shape of the input feature matrix matches that of the output feature matrix, a shortcut connection is employed, as shown in **Figure 9**. In addition, the dimensionality compression process in MobileNetv2 uses a linear activation function instead of the Relu activation function to reduce information loss caused by compression.

To reduce computing costs and memory usage, this study utilizes the first 8 layers of the MobileNetv2 model. This choice is made because starting from the 9th layer, the number of output channel increases to 1280, leading to higher computing resource consumption. To minimize the loss of down-sampling information while increasing receptive field, the stride of the 7th layer is modified to 1 (Meng et al., 2020).

Furthermore, dilated convolutions with a factor not exceeding 1 are utilized to replace conventional convolutions. According to research by Wang et al. (2018), sparse concatenation of dilated convolution may introduce grid effects, hindering the lower layers of the network from fully leveraging features from the initial layer and causing the loss of fine-grained details. Therefore, dilation rates of 2 and 5 are applied in the 7th and 8th layer respectively, while the remaining layers maintain a dilation rate of 1, aiming to expand the receptive field and preserve edge detail information. The structure and hyperparameter of the improved MobileNetv2 are displayed in **Table 4**, in which *t* is the expansion factor, *c* is the output channel, n is the number of repetitions of bottleneck, *s* is the first module strid, and *r* is dilation rate. When dilation rate of 1 results in atrous convolution being equivalent to a regular convolution. This design achieves a balance between computational resource consumption and network performance requirements.

**Table 4:** Hyperparameters of MobileNetv2.

Input size	Operator	*t*	*c*	*n*	*s*	*r*
512×512×3	conv2d	–	32	1	2	1
256×256×32	bottleneck	1	16	1	1	1
256×256×16	bottleneck	6	24	2	2	1
128×128×24	bottleneck	6	32	3	2	1
64×64×32	bottleneck	6	64	4	2	1
32×32×64	bottleneck	6	96	3	1	1
32×32×96	bottleneck	6	160	3	1	2
32×32×160	bottleneck	6	320	1	1	5

#### 2.3.3 Strip pooling

**Figure 10:**
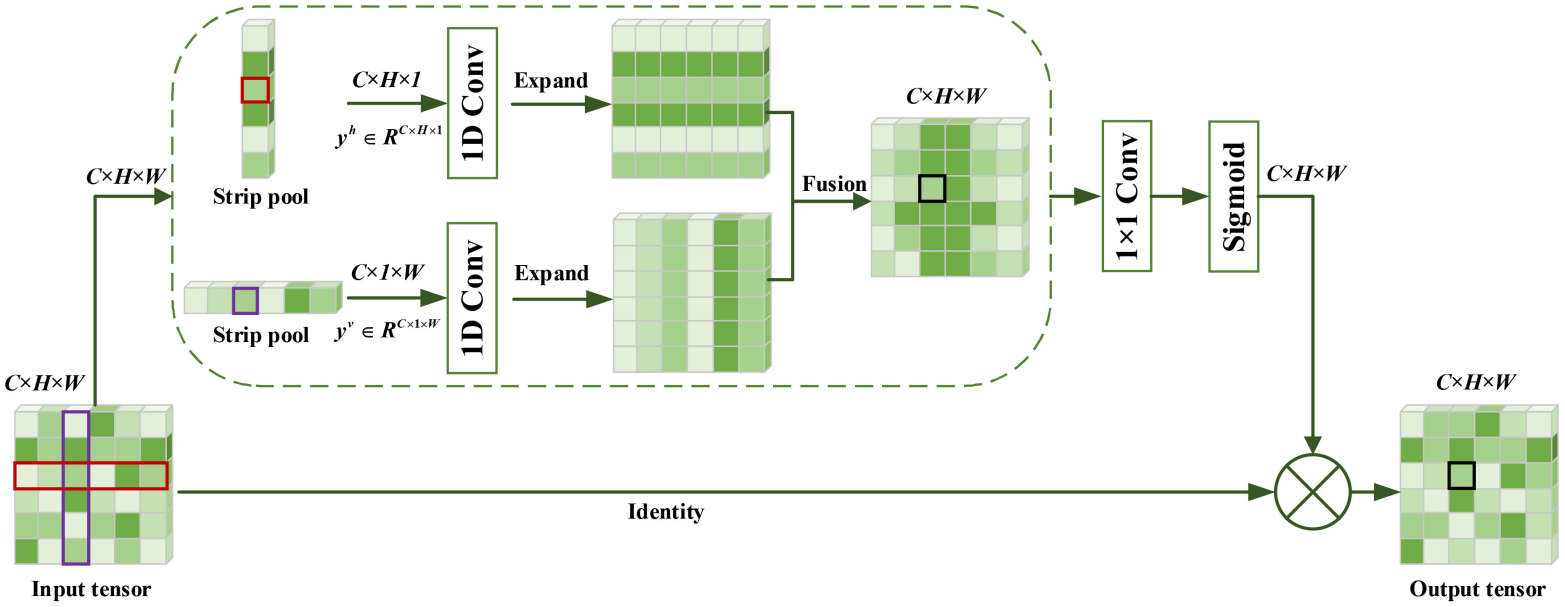
Structure of strip pooling.


To better handle the segmentation of broken cane and top with irregular and complex shapes, a lightweight strip pooling layer was added in parallel to DSC in the ASPP. This allows for more efficient acquisition of information from a large receptive field, facilitating the collection of remote contextual information from different spatial dimensions by ASPP. Strip pooling utilizes a pooling kernel (rectangular area) that performs pooling operations along the horizontal and vertical dimensions. The structure of strip pooling (Hou et al., 2020) is shown in **Figure 10**, where X ϵ *R^C^* ^×^ *^H^* ^×^ *^W^* is the input tensor, *C* denotes the number of channels, *H* denotes the height, and W denotes the width. First, the input X is pooled horizontally and vertically to obtain *y^h^* ϵ *R^C^* ^×^ *^H^* ^×^ *^1^* and *y^v^* ϵ *R^C^* ^×^ *^1^* ^×^ *^W^*, respectively. Then, the feature maps are expanded to the same resolution C×H×W as the input X using a 1D convolution with a kernel size of 3×3 to obtain the expanded *y^h^*, *y^v^*. Next, the expanded feature maps are fused to obtain a final representation.

$$y_{c,i,j}=y_{c,i}^{h}+y_{c,j}^{v},1\leq c\leq C,1\leq i\leq H,1\leq j\leq W$$

Finally, after a 1 × 1 standard convolution and a sigmoid layer, the final output Z of strip pooling is obtained by multiplying the corresponding elements with the original input.


Z=Scale(X,σ(f(y)))

where Scale (-, -) is the element-level multiplication, *σ* is the sigmoid function, and *f* is the 1×1 convolution, *y* is feature fusion results.

The element of specified location in the output tensor (*i,j*),1≤*i*≤*H*,1≤*j*≤*W* corresponds to the result of strip pooling of the horizontal and the vertical pooling window in the input tensor. By repeatedly applying the aggregation process using long and narrow pooling kernels, the ASPP_DS module can efficiently capture information from a wide receptive field throughout the entire scene. Due to the design of the elongated and narrow shape of the pooling kernel, it not only establishes remote dependency relationships between regions distributed discretely but also focuses on capturing local detailed features.

#### 2.3.4 Coordinate attention

Inspired by the prominence of the region-of-interest search in the human visual system, attention mechanisms aim to simulate this process by dynamically adjusting the weights based on the input image features. Attention mechanisms can be categorized into various types, such as channel attention (e.g. SE), hybrid attention (e.g. CBAM), temporal attention (e.g. GLTR), branch attention (e.g. SKNet), and position attention mechanisms (e.g. CA). These attention mechanisms have been widely applied in fields such as object detection (Yu J. et al., 2023) and image segmentation (Zhu et al., 2023).

**Figure 11:**
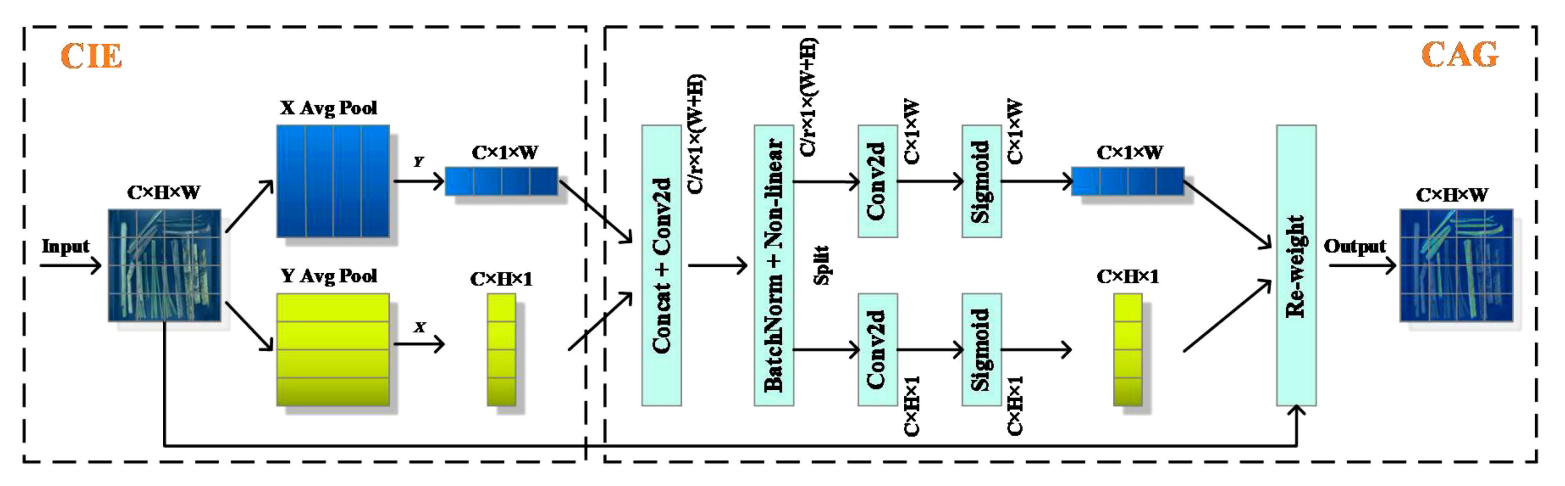
Structure of coordinate attention.

The CA not only models channel relationships but also utilizes positional information to capture long-range dependencies (Hou et al., 2021). Therefore, CA was selected in the MDSC-DeepLabv3+ to highlight the regions of interest. The CA consists of coordinate information embedding (CIE) and coordinate attention generation (CAG) two main operation, as shown in **Figure 11**. CIE introduces two global average pooling to encode each channel along the horizontal and vertical coordinate on the input feature map, respectively, hence aggregates features along the two spatial directions. These two pairs of global average pooling operation enable CA to capture long-range dependencies along one spatial direction and preserve precise positional information along other one, which allows the network to more precisely locate the objects of interest. CAG first conducts concatenation (Concat) and Conv2d for the feature maps obtained from CIE followed by batch normalization and non-linear activation operation. Then, the intermediate feature map is split into two separate tensors along the spatial dimension. Next, 1×1 Conv2d and sigmoid activation are utilized to separately transform the output tensors to tensors with the same channel number as the input feature maps. Finally, the output tensors are then expanded into elements and used as attention weights. The final output of CA is the element-wise multiplication of original input of CIE and the attention weights.

Introduction of CA before low feature processing and after the features fusion of ASPP_DS is beneficial in fully utilizing positional information. This allows the model to accurately capture the spatial relationships and contextual information of the target, thus improving the accuracy of sugarcane and impurity phenotype segmentation in denser images.

## 3 Experiments and results

### 3.1 Analyzing of estimation model

**Figure 12:**
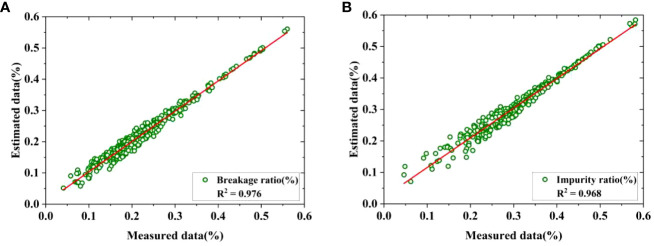
Fitting of estimated and measured ratio. **(A)** Breakage ratio, **(B)** Impurity ratio.

The effectiveness of estimation model for breakage and impurity ratios defined in Section 2.2.2 was validated by fitting estimated and measured value. First, the measured mass of cane, broken cane, top, and cane leaf *M_C_*, *M_B_*, *M_T_* and *M_L_*, along with the number of pixels for each category manually labeled in the selected 285 images (95% confidence interval of samples) were obtained. Then, estimated masses of *M'_C_*, *M'_B_*, *M'_T_* and *M'_L_* for the four categories were determined based on the mean surface density *μ_C_*, *μ_B_*, *μ_T_* and *μ_L_* according to Eq.(1)-(4). Next, the measured and estimated ratios of breakage and impurity were obtained according to Eq.(5)-(6) based on the measured and estimated masses. Finally, the measured breakage and impurity ratios were linearly fitted with the estimated breakage and impurity ratios, and the fitting results are shown in **Figure 12** and **Table 5**, respectively.

**Table 5:** ANOVA of breakage and impurity ratios.

Ratio		DF	Square sums	Mean square	F	Significance F
Breakage ratio	Regression analysis	1	2.58018	2.58018	11405.03085	1.05518E-230
	Residual	283	0.06402	2.26232E-4		
	Total	284	2.64421			
Impurity ratio	Regression analysis	1	2.41267	2.41267	8470.24579	6.21725E-213
	Residual	283	0.08061	2.84841E-4		
	Total	284	2.49328			

It can be observed that the fitting *R^2^* values are as high as 0.976 and 0.968, respectively. In addition, the results of the ANOVA presented in **Table 5** indicate a high correlation between the estimated breakage and impurity ratios and their measured values, with a significance level of *F*<0.01. Therefore, it is feasible to utilize the fitted surface density to estimate mass for each category and furthermore predict the breakage and impurity ratios for raw sugarcane.

### 3.2 Analyzing of segmentation model

#### 3.2.1 Training environment and evaluation metrics

The semantic segmentation categories considered in this study are background, cane, broken cane, top, and leaf. In the process of sugarcane harvesting, raw sugarcane is primarily composed of cane, with cane tops and leaves present as impurities to a lesser extent. Broken cane represents the category with the lowest representation, leading to an extreme class imbalance. Consequently, this often leads to imbalanced positive and negative samples, along with varying sample difficulties. Therefore, this study utilizes the Focal Loss function as the primary loss function to address the imbalance between easy and difficult samples, facilitating better parameter optimization during the backpropagation process (Lin et al., 2017). In addition, the model incorporates the multi-class Dice Loss as an auxiliary loss function to enhance segmentation accuracy and address class imbalance scenarios (Milletari et al., 2016). The combination of Focal Loss and multi-class Dice Loss as the loss function enhances the model’s predictive capability. The Focal loss for multi-objective segmentation is defined as.


$$L_{F}=-\alpha_{t}{\left(1-p_{t}\right)}^{\gamma }\log\left(p_{t}\right)$$

Where *p_t_* is the confidence value of the sample category prediction. γ is an adjustable parameter, and the default is 2.

The Dice loss for multi-objective segmentation is defined as.


$$L_{D}=1-\sum_{j=1}^{c}\frac{2W_{j}\sum_{i=1}^{N}gt(j,i)\log(p_{i,j})}{\sum_{i=1}^{N}\left(gt{\left(j,i\right)}^{2}+\log{\left(p_{i,j}\right)}^{2}\right)}$$

Where, *N* is the number of samples, *c* is the target class, and *p_i,j_* is the softmax output of class *j* target class; *gt*(*j,i*) is the ground-truth label of class *j* target, and *W_j_* is the weight of the objective of class *j*, *W_j_* = *1/j*.

The experiments were conducted on a server in the lab with the configuration shown in **Table 6**. The MDSC-DeepLabv3+ used the Adam optimizer to compute the gradient of the loss function in each epoch to perform parameter updates. The initial learning rate was set to E-4. The batch size was set to 6. The training process consists of 100 epochs. In each epoch, the image dataset was randomly shuffled and fed into the model to ensure a different order of dataset used in different epochs. This technique enhances the convergence speed of the model and improves the prediction results on the test set.

**Table 6:** Experimental environment.

Parameter	Configuration	Parameter	Configuration
Operating system	Ubuntu 18.04	Operating environment	CUDA 11.2
Deep learning framework	PyTorch 1.8	CPU	Intel(R) Xeon(R) Silver 4214 CPU @2.20GHz
Programming Language	Python 3.7	GPU	NVIDIA GeForce RTX 3080 12G @1260-1710MHz

In order to comprehensively evaluate the performance of the proposed and comparative semantic segmentation models, three aspects of each model, namely accuracy, deployability, and efficiency, are comprehensively evaluated. The commonly used mIoU and mPA were utilized as accuracy evaluation metrics. And the model deployability was evaluated using model parameter quantity (Param) and model computation volume floating point operations (FLOPs). Efficiency was evaluated using inference time for each image. The metrics of IoU, mIoU and mPA which is represented by the following Eq. (7)-(9), respectively.


(7) $$IoU_{i}=\frac{P_{ii}}{\sum_{i=0}^{c-1}P_{ij}+\sum_{j=0}^{c-1}P_{ji}-P_{ii}}\times 100\%$$

(8) $$mIoU=\frac{1}{c}\sum_{i=0}^{c-1}IoU_{i}$$

(9) $$mPA=\frac{1}{c}\sum_{i=0}^{c-1}\frac{p_{ii}}{\sum_{j=0}^{c-1}p_{ij}}$$

Where *c* denotes the number of categories, so *c*=4 (cane, broken cane, top and leaf), *P_ij_* or *P_ji_* denotes the number of category prediction that is incorrect, while *P_ii_* denotes the number of correct predictions made by categories.

#### 3.2.2 Model training

The size of the input image is a crucial factor affecting the model’s performance. Increasing the image size enhances accuracy by preserving semantic information for small targets and preventing information loss caused by low-resolution feature maps. However, excessively large image sizes can lead to reduced detection accuracy due to the limited receptive field imposed by the fixed network structure. This, in turn, diminishes the network’s ability to accurately predict targets of various scales (Lin et al., 2022). In practical applications, there is a trade-off between accuracy and speed that requires careful consideration. For this study, the input image was resized to three different dimensions: 256×256, 512×512, and 768×768. The proposed MDSC-DeepLabv3+ model was trained accordingly, and the results obtained are presented in **Table 7**. It can be observed that reducing the input image size to 512×512 achieves an optimal balance between speed and accuracy.

**Table 7:** mPA and inference time obtained with different input image sizes.

Resize of image/pixels	mPA/%	Inference time/ms
256×256	94.68	10.69
512×512	97.55	13.85
768×768	97.07	24.19

**Figure 13:**
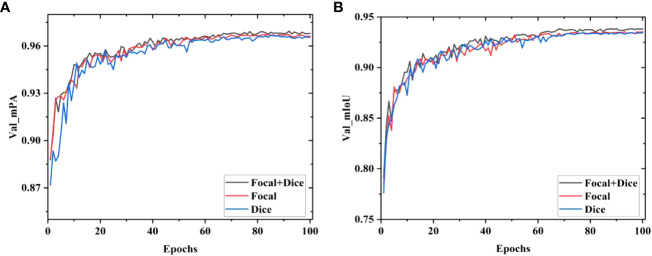
Results of mPA and mIoU with different loss functions. **(A)** Valid mPA, **(B)** Valid mIoU.

The segmentation results of models using different loss functions are displayed in **Figure 13**. The MDSC-DeepLabv3+ using only the Dice loss function exhibits the highest fluctuations in mPA and mIoU, leading to inferior segmentation results. Similarly, the MDSC-DeepLabv3+ using only Focal Loss demonstrates notable fluctuations during the early stages of the validation process, with slow growth in mPA and mIoU values in later stages. In contrast, the MDSC-DeepLabv3+ which combines Focal Loss and multi-class Dice Loss exhibits lesser sawtooth fluctuations during the increase in mPA and mIoU values, ultimately reaching their peak during the validation process. Consequently, the integration of Focal Loss and multi-class Dice Loss yields optimal outcomes in the segmentation of raw sugarcane and impurities.

#### 3.2.3 Ablation experiment

To verify the effectiveness of the three improvements, including improved MobileNetv2, ASPP_DS and CA presented in Section 2.3, the following 7 models were constructed according to the control variable method, with a downsampling factor of 8.

1. DeepLabv3+_base: MobileNetv2 replaced the backbone Xception in DeepLabv3+.
2. M-DeepLabv3+: MobileNetv2 in DeepLabv3+_base was enhanced with atrous convolution operation.
3. MDS-DeepLabv3+: ASPP_DS replaced ASPP module in M-DeepLabv3+.
4. MC1-DeepLabv3+: CA was applied independently before 1×1 Conv of low-level features by the decoder in M-DeepLabv3+.
5. MC2-DeepLabv3+: CA was applied independently after the fusion of ASPP in M-DeepLabv3+.
6. MC-DeepLabv3+: CA was added separately before 1×1 Conv the low-level features and after the fusion of ASPP features in M-DeepLabv3+.
7. MDSC-DeepLabv3+: CA was added separately before processing the low-level features and after the fusion of ASPP_DS features in MDS-DeepLabv3+.

**Table 8** presents the results of the ablation experiment for the seven aforementioned models. It can be observed that the MDSC-DeepLabv3+ outperforms the baseline DeepLabv3+_base, with an improvement of 1.25 in mPA and 1.8 in mIoU. Additionally, it achieves a reduction of 16.42% in Params and 31.46% in FLOPs, however, the inference time per image has slightly increased from 13.48ms to 13.85ms. These results demonstrate that the MDSC-DeepLabv3+ surpasses the DeepLabv3+_base in terms of segmentation accuracy and deployability metrics, while still maintaining comparable efficiency. Furthermore, it can be seen that the MDSC-DeepLabv3+ achieves the highest segmentation accuracy (mPA and mIoU) compared to other models, while exhibiting minimal differences in terms of deployability (Params, FLOPs) and efficiency (inference time) metrics.

**Table 8:** Results of ablation experiment.

Number	ASPP_DS	Coordinate Attention		mPA/%	mIoU/%	Param/M	FLOPs/G	Inference time/ms
		Before decoder	After ASPP(_DS)					
(1)				96.3	93.04	4.81	69.29	13.48
(2)				96.67	93.36	3.35	45.49	12.13
(3)	√			97.16	94.37	3.36	45.41	12.76
(4)		√		96.88	93.66	3.55	46.83	13.51
(5)			√	97.05	94.48	3.63	46.88	13.56
(6)		√	√	97.22	94.57	3.68	46.88	13.67
(7)	√	√	√	97.55	94.84	4.02	47.49	13.85

**Figure 14:**
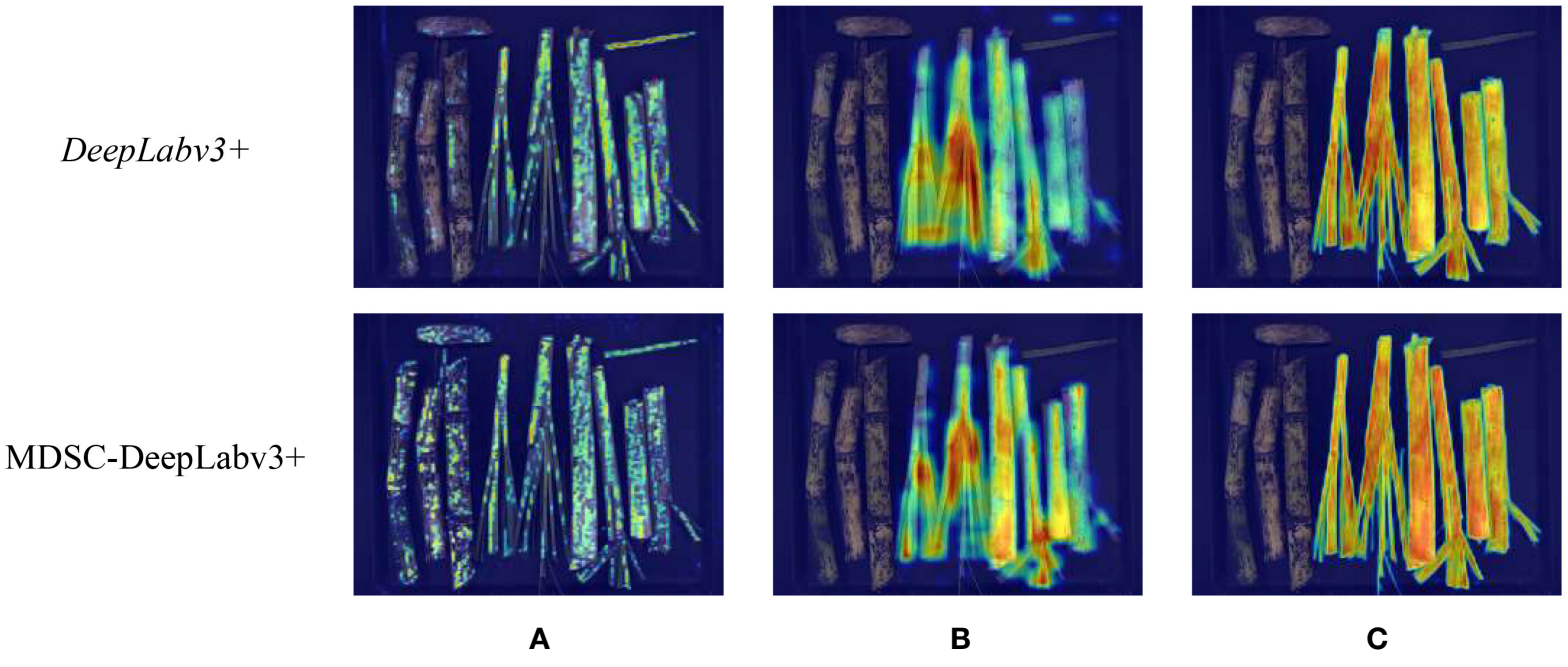
**(A)** Backbone output. **(B)** Encode output. **(C)** Decode output.

In order to visually demonstrate the improvement of the models, Grad-CAM (Selvaraju et al., 2020) was used to visualize the channels of the feature maps of DeepLabv3+ and MDSC-DeepLabv3+. The visualization segmentation instances of top were illustrated in **Figure 14**. In group (a), the two feature maps are extracted by the Xception in DeepLabv3+ and the enhanced MobileNetv2 in MDSC-DeepLabv3+, respectively. In group (b), two feature maps are the output of ASPP in DeepLabv3+ and ASPP_DS in MDSC-DeepLabv3+, respectively. In group (c), the two feature maps are the output of DeepLabv3+ and MDSC-DeepLabv3+, respectively.

In **Figure 14A**, it can be observed that Xception in DeepLabv3+ achieves clearer pixel segmentation than that obtained by MobileNetv2 in MDSC-DeepLabv3+. The reason is that MobileNetv2 is a lightweight and shallow model compared to Xception, and its depthwise convolution can lead to information loss and limit the number of channels, thereby resulting in a lower-level feature map with fewer information. However, the two heat maps in group (b) indicate that there is pixels misfocus at the top-right corner in the first line of the feature map extracted by ASPP, while ASPP_DS results in more complete pixel segmentation, enhances preservation of details, and eliminates the top-right misfocus. The heat map illustrates that the introduced strip pooling in ASPP_DS rectifies the shortage of MobileNetv2, and the dense and compact dilation rates (4, 8, 12) improve its capability of focusing on capturing local detailed features. Heat map of final outputs of MDSC-DeepLabv3+ and DeepLabv3+ given in **Figure 14C** demonstrates that the CA in MDSC-DeepLabv3+ further enhances the color intensity in heat map, indicating that the inclusion of CA allows the model to focus more on the features of the categories, thereby enhancing its distinguishability of cane, broken cane, top and leaf.

#### 3.2.4 Comparative experiment

To further validate the superiority of the proposed model MDSC-DeepLabv3+, comparative experiments were conducted using the RSI dataset under the same experimental conditions. The compared models include UNet, PSPNet, SegFormer-B0, and the baseline DeepLabv3+. Previous research results have shown that UNet (Ronneberger et al., 2015) and PSPNet (Zhao et al., 2017) perform well in terms of accuracy in segmentation tasks with challenges like cell tracking ISBI and Cityscapes. SegFormer-B0 is a lightweight model that combines transformers with a lightweight multilayer perceptron decoder (Xie et al., 2021). The comparative results are given in **Table 9**.

**Table 9:** Test results of different recognition models.

Segmentation models	IoU/%					mIoU/%	mPA%	Param/M	FLOPs/G	Inference time/ms
	Background	Cane	Broken cane	Top	Leaf					
UNet	98.13	94.18	91.01	93.11	93.73	94.03	96.86	24.89	451.77	27.14
PSPNet	95.45	90.38	87.89	86.48	87.89	89.62	94.85	46.71	118.43	15.42
SegFormer-B0	95.6	82.98	72.38	81.12	79.79	82.37	89.79	3.72	13.56	16.78
DeepLabv3+	97.78	95.18	91.83	93.38	94.62	94.56	97.21	42.19	141.22	24.43
MDSC-DeepLabv3+	97.94	95.13	91.85	94.27	95.03	94.84	97.55	4.07	47.49	13.85

It can be seen that the accuracy of MDSC-DeepLabv3+ surpasses that of the aforementioned four models with significant improvements. Specifically, the mIoU of MDSC-DeepLabv3+ is higher by 0.81, 5.22, 12.47, and 0.28 compared to UNet, PSPNet, SegFormer-B0, and DeepLabv3+, respectively. Moreover, the mPA of MDSC-DeepLabv3+ reaches an impressive 97.55%, which outperforms UNet, PSPNet, SegFormer-B0, and DeepLabv3+ by 0.69, 2.7, 7.76, and 0.34, respectively. These remarkable improvements can be attributed to the adoption of the advanced DeepLabv3+ as the basic model, coupled with the enhancements introduced through strip pooling and CA. Strip pooling plays a crucial role in collecting remote contextual information from different spatial dimensions and addressing the issue of information loss resulting from the atrous convolution operation in DeepLabv3. On the other hand, CA efficiently utilizes positional information, enabling accurate capturing of the spatial relationships and contextual information of the detected cane, broken cane, top, and leaf.

In terms of deployability, MDSC-DeepLabv3+ demonstrates remarkable reductions in Params and FLOPs when compared to UNet, PSPNet, and DeepLabv3+. Specifically, it reduces Params by 83.65%, 91.29%, and 90.35%, and FLOPs by 89.49%, 59.9%, and 66.37% compared to UNet, PSPNet, and DeepLabv3+ respectively. This significant reduction in model size and computational complexity makes MDSC-DeepLabv3+ highly efficient and resource-friendly. Moreover, MDSC-DeepLabv3+ achieves impressive segmentation efficiency, with a recognition speed of only 13.85ms per image. This inference time per image is far less than the above three models, with reductions of 48.97%, 10.18%, and 43.31%, respectively. This indicates that MDSC-DeepLabv3+ is able to perform fast and accurate segmentation, making it highly suitable for real-time applications. Although SegFormer-B0 may have some advantages in terms of deployability, its accuracy is much lower compared to MDSC-DeepLabv3+ (89.79% vs. 97.55%). The reason for this superior performance is the utilization of the improved lightweight MobileNetv2, which replaces Xception in DeepLabv3+, leading to an efficient and accurate model overall. In summary, the proposed MDSC-DeepLabv3+ outperforms the compared four models in the task of segmenting sugarcane and impurities, offering a winning combination of high segmentation accuracy, deployability, and recognition speed.

**Figure 15:**
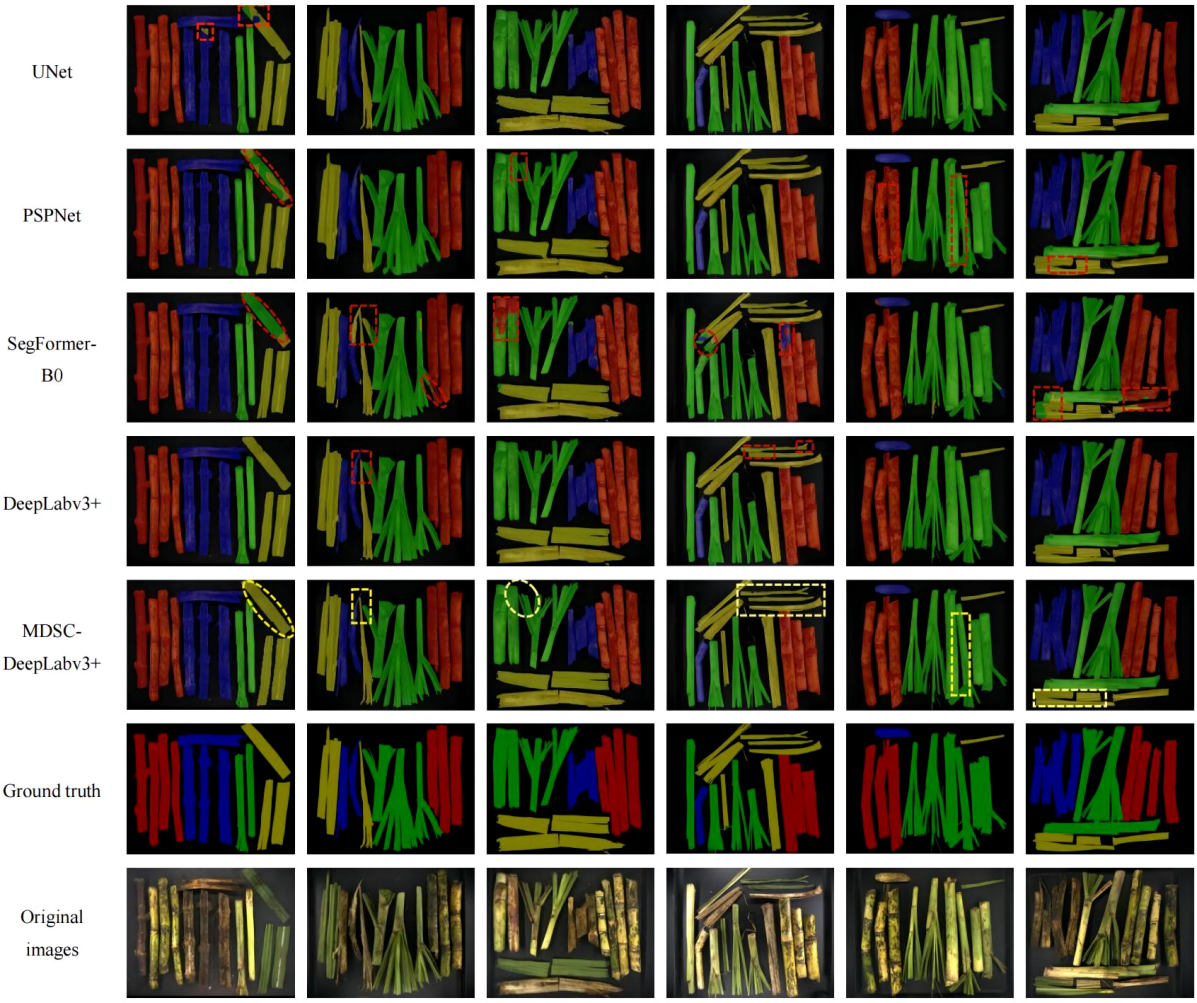
Test results of each detection model.

Instances of the results obtained using the aforementioned segmentation models are illustrated in **Figure 15**. In which, red [128,0,0] represents cane, blue [0,0,128] represents broken cane, green [0,128,0] represents top, yellow [128,128,0] represents leaf, and black [0,0,0] represents the background. From the visualization of test results, it is evident that all five models perform well in most cases. However, the segmentation obtained by MDSC-DeepLabv3+ stands out as more complete, with clearer preservation of details in general. Upon closer observation, it can be seen that UNet, PSPNet, and SegFormer-B0 misclassify their categories, for instance, misclassifying broken cane as leaf, and vice versa. This indicates inaccuracies in pixel differentiation for these models. Additionally, the compared four models result in fuzzy segmentation and ambiguous boundaries between objects. On the other hand, the proposed MDSC-DeepLabv3+ demonstrates superior performance in addressing the issue of detail adhesion. This can be observed in the instances marked out in the line of MDSC-DeepLabv3+ where the model is capable of better distinguishing object boundaries and preserving fine details.

### 3.3 Analyzing of comprehensive experiment

The breakage and impurity ratios of raw sugarcane were estimated using the estimation model presented in Section 2.2 and the MDSC-DeepLabv3+ segmentation model presented in Section 2.3. These estimated values were then compared with the measured breakage and impurity ratios obtained through manual weighing to assess the effectiveness of the proposed method.

First, a subset of 25% (70) of the images was randomly selected from the mass dataset with 300 samples. The MDSC-DeepLabv3+ model was applied to semantically segment the selected 70 images and determine the number of cane, broken cane, top, and leaf pixels for each image. Then, corresponding masses were estimated using Eq.(1)-(4), based on the mean values of the surface density for each category obtained through normal fitting. The ratios of breakage and impurity were calculated according to the estimation model defined in Eq.(5)-(6) based on the estimated masses. Finally, the measured breakage and impurity ratios were determined using the measured mass and the relative errors between the estimated and measured results were calculated. **Tables 10**. **11** document and analyze the relative errors in the breakage ratio and impurity ratio for each sample, as well as the average relative error of the overall samples. The average relative errors were found to be 11.3% and 6.5% for breakage and impurity ratios, respectively. These results indicated that the proposed method exhibits strong reliability.

**Table 10:** Breakage ratios of 70 samples.

Sample number	Breakage ratio/%			Sample number	Breakage ratio/%		
	Measured	Estimated	Relative errors		Measured	Estimated	Relative errors
1	0.393	0.433	0.103	36	0.163	0.146	0.103
2	0.244	0.215	0.119	37	0.067	0.077	0.148
3	0.328	0.319	0.027	38	0.075	0.077	0.017
4	0.122	0.096	0.218	39	0.117	0.127	0.082
5	0.259	0.272	0.049	40	0.253	0.242	0.047
6	0.486	0.562	0.156	41	0.381	0.418	0.097
7	0.319	0.290	0.090	42	0.145	0.125	0.137
8	0.165	0.174	0.057	43	0.268	0.259	0.036
9	0.173	0.201	0.162	44	0.272	0.247	0.091
10	0.298	0.269	0.097	45	0.060	0.046	0.231
11	0.389	0.416	0.069	46	0.298	0.323	0.087
12	0.235	0.284	0.208	47	0.192	0.168	0.126
13	0.225	0.222	0.012	48	0.209	0.209	0.001
14	0.102	0.131	0.282	49	0.361	0.343	0.049
15	0.105	0.141	0.340	50	0.112	0.150	0.344
16	0.152	0.163	0.077	51	0.233	0.193	0.171
17	0.403	0.340	0.157	52	0.226	0.215	0.048
18	0.144	0.154	0.071	53	0.253	0.281	0.110
19	0.108	0.124	0.150	54	0.299	0.271	0.093
20	0.273	0.267	0.025	55	0.056	0.071	0.262
21	0.388	0.404	0.042	56	0.138	0.168	0.218
22	0.371	0.387	0.045	57	0.141	0.167	0.188
23	0.456	0.480	0.052	58	0.109	0.106	0.035
24	0.264	0.247	0.064	59	0.201	0.207	0.028
25	0.348	0.330	0.053	60	0.385	0.425	0.105
26	0.257	0.240	0.065	61	0.314	0.289	0.079
27	0.170	0.136	0.198	62	0.120	0.130	0.089
28	0.184	0.149	0.191	63	0.227	0.201	0.113
29	0.353	0.337	0.044	64	0.125	0.120	0.044
30	0.351	0.343	0.023	65	0.416	0.451	0.084
31	0.296	0.255	0.138	66	0.160	0.195	0.219
32	0.356	0.342	0.039	67	0.281	0.278	0.011
33	0.214	0.233	0.088	68	0.162	0.186	0.149
34	0.215	0.277	0.286	69	0.322	0.277	0.141
35	0.172	0.150	0.132	70	0.195	0.231	0.184
				Average			0.113

**Table 11:** Impurity ratios of 70 samples.

Sample number	Impurity ratio/%			Sample number	Impurity ratio/%		
	Measured	Estimated	Relative errors		Measured	Estimated	Relative errors
1	0.473	0.499	0.055	36	0.328	0.319	0.026
2	0.237	0.254	0.071	37	0.313	0.322	0.032
3	0.423	0.424	0.004	38	0.091	0.104	0.142
4	0.292	0.298	0.021	39	0.217	0.240	0.103
5	0.303	0.263	0.133	40	0.380	0.381	0.001
6	0.602	0.570	0.053	41	0.241	0.240	0.005
7	0.445	0.445	0.002	42	0.369	0.369	0.000
8	0.372	0.341	0.082	43	0.292	0.282	0.035
9	0.393	0.352	0.104	44	0.328	0.337	0.026
10	0.294	0.280	0.048	45	0.146	0.167	0.148
11	0.529	0.554	0.046	46	0.274	0.316	0.156
12	0.277	0.272	0.018	47	0.310	0.273	0.118
13	0.378	0.388	0.028	48	0.410	0.382	0.068
14	0.206	0.199	0.034	49	0.254	0.269	0.063
15	0.332	0.314	0.055	50	0.320	0.319	0.004
16	0.240	0.217	0.098	51	0.343	0.386	0.124
17	0.482	0.452	0.062	52	0.328	0.325	0.009
18	0.277	0.298	0.073	53	0.385	0.355	0.077
19	0.331	0.317	0.043	54	0.211	0.232	0.102
20	0.274	0.265	0.034	55	0.228	0.248	0.088
21	0.358	0.322	0.102	56	0.420	0.389	0.073
22	0.491	0.470	0.042	57	0.268	0.267	0.007
23	0.417	0.439	0.054	58	0.209	0.200	0.043
24	0.286	0.318	0.110	59	0.239	0.245	0.023
25	0.273	0.241	0.119	60	0.427	0.421	0.014
26	0.316	0.337	0.066	61	0.332	0.320	0.036
27	0.267	0.265	0.006	62	0.319	0.315	0.011
28	0.251	0.272	0.082	63	0.253	0.239	0.054
29	0.208	0.249	0.196	64	0.313	0.339	0.082
30	0.375	0.334	0.109	65	0.500	0.483	0.034
31	0.296	0.347	0.173	66	0.418	0.378	0.095
32	0.229	0.290	0.265	67	0.297	0.320	0.077
33	0.283	0.279	0.014	68	0.299	0.337	0.128
34	0.357	0.332	0.072	69	0.475	0.465	0.021
35	0.350	0.342	0.024	70	0.301	0.302	0.002
				Average			0.65

**Figure 16:**
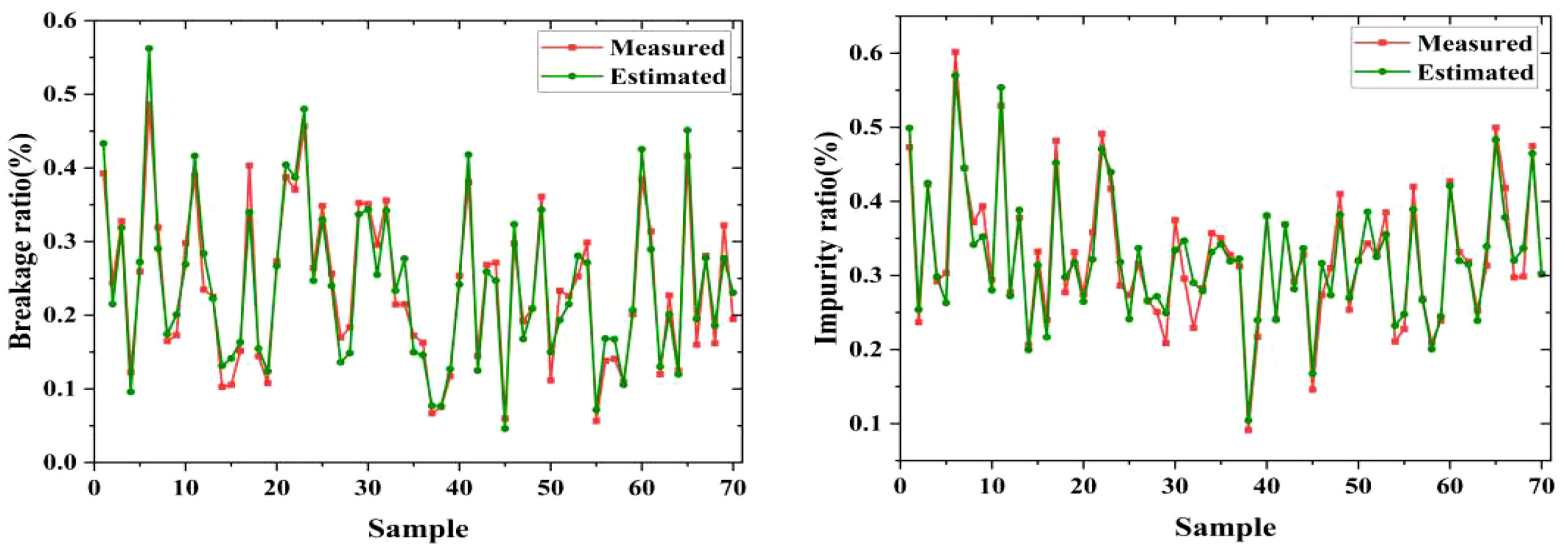
Instances of estimation and measured breakage and impurity ratio.

Additionally, the visualization of measured and estimated ratios of the 70 samples is depicted in **Figure 16**. This aids in the intuitive observation and analysis of the relationship and differences between predicted and manual measured results. It can be observed that the results obtained using the proposed method exhibit only slight deviations compared to the results obtained through manual weighing measurements, and the fluctuations are minimal. This suggests that the estimated breakage and impurity ratios can maintain their stability. Consequently, the proposed method based on estimation model and MDSC-DeepLabv3+ offers an efficient, accurate, and intelligent means of quantitatively estimating the breakage and impurity ratios of raw sugarcane.

## 4 Conclusions

In practice, objective, efficient, accurate, and intelligent detection of breakage and impurity ratios is an urgent requirement in the sugar refinery. Therefore, this study developed a novel approach combining the estimation model and MDSC-DeepLabv3+ segmentation network to tackle this problem. First, a machine vision-based acquisition platform was designed, and custom image and mass datasets of raw sugarcane and impurities were constructed. Then, estimation model was built to assess the ratios of breakage and impurity, considering the variation of surface density for the four categories of objects. Finally, the MDSC-DeepLabv3+ segmentation network dedicated to the detection of cane, broken cane, top, and leaf was developed. It effectively incorporated improved MobileNetv2, ASPP_DS, and CA based on DeepLabv3+ to enhance segmentation accuracy, reduce parameters and inference time. The analysis of the experimental results leads to the following conclusions:

1. The breakage and impurity ratios obtained through estimation model based on normal fitted surface density exhibit high accuracy, with corresponding *R^2^* of 0.976 and 0.968, respectively.
2. The proposed MDSC-DeepLabv3+ achieved superiority considering segmentation accuracy, deployability, and efficiency simultaneously. The mPA and mIoU achieved by MDSC-DeepLabv3+ were as high as 97.55% and 94.84%, respectively, surpassing the baseline DeepLabv3+ by 0.34 and 0.28. This improvement in accuracy was accomplished with 38.12M, 93.73G, and 10.58ms reduction in Params, FLOPs, and inference time, respectively, making it advantageous for deployment on edge devices and real-time inference.
3. The estimated data obtained according to the approach developed in this study fit the manually obtained breakage and impurity ratios with average relative errors of 11.3% and 6.5%, respectively. The lower segmentation accuracy of broken *cane* is due to their burr and ambiguous boundaries, resulting in a higher average relative error of the breakage ratio.

The raw sugarcane not only includes top and leaf impurities but also contains other impurities like dispersed root whiskers. The upcoming research will emphasize mechanical cleaning of sand, gravel, soil, and similar substances. Additionally, a pivotal aspect of the forthcoming study will involve counting sugarcane roots and estimating their quality through object detection.

## Data availability statement

The raw data supporting the conclusions of this article will be made available by the authors, without undue reservation.

## Author contributions

XL: Methodology, Writing – original draft, Writing – review & editing. ZZ: Funding acquisition, Methodology, Writing – review & editing. SL: Methodology, Writing – review & editing. TL: Data curation, Investigation, Visualization, Writing – original draft. JZ: Data curation, Visualization, Writing – original draft. TN: Visualization, Writing – original draft. CJ: Investigation, Writing – original draft.
